# Dr. Horace Doolittle

**Published:** 1915-11

**Authors:** 


**Dr. Horace Doolittle,** the oldest practicing dentist in the state, died at his home in Albion, October 20, aged 80.

